# MARCKSL1 interacted with F‐actin to promote esophageal squamous cell carcinoma mobility by modulating the formation of invadopodia

**DOI:** 10.1002/cam4.5079

**Published:** 2022-07-27

**Authors:** Yue Zhao, Xiufeng Xie, Lusong Tian, Fang Liu, Yulin Sun, Haizhen Lu, Xiaohang Zhao, Yousheng Mao

**Affiliations:** ^1^ Department of Thoracic Surgery China‐Japan Friendship Hospital Beijing China; ^2^ Department of Thoracic Surgery National Cancer Center/National Clinical Research Center for Cancer/Cancer Hospital, Chinese Academy of Medical Sciences and Peking Union Medical College Beijing China; ^3^ State Key Laboratory of Molecular Oncology National Cancer Center/National Clinical Research Center for Cancer/Cancer Hospital, Chinese Academy of Medical Sciences and Peking Union Medical College Beijing China; ^4^ Department of Pathology National Cancer Center/National Clinical Research Center for Cancer/Cancer Hospital Chinese Academy of Medical Sciences and Peking Union Medical College Beijing China

**Keywords:** esophageal cancer, invadopodia formation, MARCKSL1, metastasis

## Abstract

**Background:**

Emerging evidence indicates that myristoylated alanine‐rich C kinase substrate like 1 (MARCKSL1) is involved in the progression of esophageal squamous cell carcinoma (ESCC). However, the underpinning mechanism is unclear. Here, we investigated the mechanisms involving MARCKSL1 in ESCC progression.

**Methods:**

CCK8, Transwell and wound‐healing assays were employed to test the effect of MARCKSL1 on proliferation, invasion and migration in vitro. Next, transcriptome profiling was conducted through RNA sequencing to reveal the underlying mechanism of MARCKSL1 in ESCC progression, which was subsequently verified by western blot and qPCR analysis. Moreover, immunofluorescence and gelatin degradation assays were performed to reveal the ability of MARCKSL1 to mediate invadopodia formation and extracellular matrix (ECM) degradation. Finally, the correlation between MARCKSL1 and the clinicopathological features of ESCC patients was assessed based on TCGA database analysis and immunohistochemistry staining of tissue microarrays.

**Results:**

Knockdown of MARCKSL1 markedly attenuated the cell motility capacity of ESCC cells in vitro, while MARCKSL1 overexpression had the opposite effect. Transcriptomic analysis showed that MARCKSL1 mediated the mobility and migration of ESCC cells. In addition, overexpression of MARCKSL1 increased the colocalization of F‐actin and cortactin at the frontier edge of migrating cells and ECM degradation. Furthermore, in ESCC patients, the mRNA level of MARCKSL1 in esophageal carcinomas (*n* = 182) was found to be notably higher than that in adjacent esophageal epithelia (*n* = 286) and the expression levels of MARCKSL1 in the tumor tissues (*n* = 811) were significantly increased compared to those in noncancerous esophageal tissues (*n* = 442) with a large sample size. Higher expression of MARCKSL1 was positively correlated with lymph node metastasis and associated with worse survival rates of patients with ESCC.

**Conclusion:**

MARCKSL1 promotes cell migration and invasion by interacting with F‐actin and cortactin to regulate invadopodia formation and ECM degeneration. High MARCKSL1 expression is positively correlated with poor prognosis in ESCC patients with lymph node metastasis.

## Graphical abstract

High MARCKSL1 expression is positively correlated with poor prognosis in esophageal squamous cell carcinoma (ESCC) patients with lymph node metastasis. MARCKSL1 promotes ESCC cells migration and invasion by interacting with F‐actin and cortactin to regulate invadopodia formation and ECM degeneration. MARCKSL1 may be a new biomarker and therapeutic target for esophageal cancer.

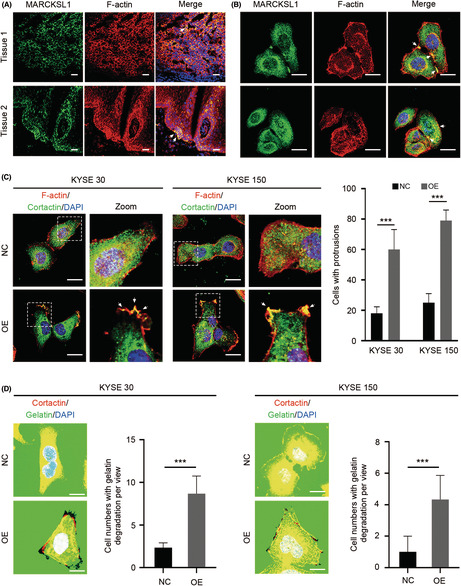

## 1 INTRODUCTION

The incidence of esophageal cancer ranks eighth worldwide, and it is also the sixth leading cause of cancer‐related mortality. Among them, esophageal squamous cell carcinoma (ESCC) is the most prevalent type.^1^ Despite advances in early detection, surgical techniques and chemoradiotherapy methods, the prognosis for ESCC remains poor, and the 5‐year survival rate is 15%–25%.^2^ The prognosis of patients with lymph node metastases is often worse than that of patients without metastases. The number and proportion of metastatic lymph nodes have been shown to be important prognostic factors.^3^ The tumorigenesis and progression of ESCC comprise a complicated, multistep process involving complex pathways and mechanisms that have not been clearly illuminated. Therefore, it is imperative to discover applicable biomarkers and novel therapeutic targets in clinical management to improve survival outcomes.

Myristoylated alanine‐rich C kinase substrate like protein 1 (MARCKSL1) is a member of the myristoylated alanine rich C kinase substrate (MARCKS) family,^4^ which bundles and stabilizes the actin strand upon phosphorylation, increasing filopodia dynamics.^5^ It has been reported that MARCKSL1 coordinates cytoskeletal organization at cell–cell and cell–substrate contacts in epithelial cells and might be relevant to cell motility.^6^ Under physiological conditions, MARCKSL1 regulates filopodium and lamellipodium formation, actin homeostasis, cell adhesion and neuronal migration, and it is critically functional in the early development of the nervous system.^4^, ^7^, ^8^, ^9^, ^10^ In addition, MARCKSL1 has also been implicated in the progression of several cancers, e.g., breast cancer,^11^ prostate cancer^5^, ^12^ and hepatocellular carcinoma,^13^ and it has been proven to be a prognosticator in certain types of patients.^14^ Emerging evidence indicates that MARCKSL1 is involved in ESCC progression,^15^ but the underlying mechanism of MARCKSL1 in ESCC progression is not fully understood.

Here, we show that upregulation of MARCKSL1 markedly promoted cell migration and invasion. The differentially expressed genes after knockdown of MARCKSL1 were significantly related to cell motility and migration through RNA‐sequencing analysis. Furthermore, colocalization of MARCKSL1 and F‐actin was further confirmed, and the results demonstrated that overexpression of MARCKSL1 accelerated invadopodia formation and extracellular matrix (ECM) degradation. In addition, MARCKSL1 was found to be significantly overexpressed in tumor tissues compared to adjacent nontumorous tissues. High expression of MARCKSL1 was positively correlated with lymph node metastasis and associated with worse patient survival rates in ESCC. MARCKSL1 may be a new biomarker and therapeutic target for esophageal cancer.

## 2 MATERIALS AND METHODS

### 2.1 Cells and treatment

All cell lines (KYSE30, KYSE510, KYSE170, KYSE150 and KYSE140) were gifts from Dr. Shimada (Hyogo College of Medicine, Japan). The cells were cultured in 10% fetal bovine serum (HyClone, Logan, UT, USA)‐RPMI 1640 medium at 37°C with 5% CO_2_. Cells were authenticated via analysis of short tandem repeat and tested negative for mycoplasma.

### 2.2 Recombinant plasmid and transfection

The cDNA of human MARCKSL1 (NM_023009) was subcloned into pcDNA3.1 to make the recombinant pcDNA3.1‐MARCKSL1 construct, which was transfected into cells by Lipofectamine 3000 (Invitrogen, Carlsbad, CA). Three siRNAs targeting human MARCKSL1 (siRNA‐1: 5′‐GGCCAACGGCCAGGAGAAUTT‐3′; siRNA‐2: 5′‐UCUCUUUCAAGAAGCCU UUTT‐3′; and siRNA‐3: 5′‐GGCUAGUGCAGCCUCAGAATT‐3′) were synthesized separately. Then, 5 × 10^5^ cells were cultured in a 6‐well plate to 60% confluence and transfected with 100 pM specific siRNA by Lipofectamine 2000 (Invitrogen).

### 2.3 Immunohistochemistry of ESCC tissues

Nine tissue microarrays (TMAs) containing 773 ESCC tissue samples and 442 corresponding normal esophageal epithelia (Outdo Biotech and Superbiotek, Shanghai, China) and 50 ESCC tissue sections collected from xxx Hospital were stained as described previously.^16^ Briefly, the tissue sections were incubated overnight at 4°C with anti‐MARCKSL1 antibody (Cat# sc‐130,471, 1:50 diluted, Santa Cruz Biotechnology, CA, USA) and then detected by a Polink‐2 Plus HRP rabbit polymer (Cat# pv‐6001, Golden Bridge, China). The results were evaluated and scored by two senior pathologists independently. Quantification was based on the percentage and the intensity of staining. The MARCKSL1 staining area scores (0, <5%; 1, 5–25%; 2, 26–50%; 3, 51–75%, and 4, ≥75%) and intensity scores (0, negative; 1, weak; 2, moderate, and 3, strong) were multiplied to generate a total score; a total score ≤6 was considered low expression and >6 was considered high expression of MARCKSL1.

### 2.4 Western blotting

Cells were lysed with RIPA buffer (150 mM NaCl, 50 mM Tris–HCl, 1.0% NP‐40, 0.5% sodium deoxycholate and 0.1% SDS) with protease inhibitor cocktail and phosphatase inhibitor (Roche). Western blotting was performed as described previously.^16^ Total protein (25–40 μg/well) was fractionated by 4%–20% gradient SDS–PAGE gels and transferred to PVDF membranes. After blocking in 5% nonfat dry milk in PBST, the membranes were incubated with primary antibodies (anti‐MARCKSL1, sc‐130,471, Santa Cruz; anti‐β‐actin, A5316, Sigma–Aldrich) at 4°C overnight. The proteins were detected with horseradish peroxidase (HRP)‐conjugated secondary antibodies and visualized with Renaissance Plus Reagent (Cat# 93702, CTS).

### 2.5 RNA extraction and quantitative PCR

RNA isolation, retrotranscription to cDNA, and qPCR (RT–qPCR) were performed as described previously.^17^ Briefly, total RNA was extracted from ESCC cells by TRIzol reagent (Invitrogen) and qPCR assays were conducted using the following primers: MARCKSL1: forward 5′‐TATCCCCCAAGGGTGAAGGG‐3′, reverse 5′‐TTCTCTTGAAGGACAGGCCG‐3′; GAPDH: forward 5′‐GGAGCGAGATCCCTCCAAAAT‐3′, reverse 5′‐GGCTGTTGTCATACTTCTCATGG‐3′. The initial denaturation was performed for 30 s at 95°C, followed by denaturing for 5 s at 95°C, annealing primers for 30 s at 60°C, extending DNA for 15 s at 95°C, and the step was repeated for 40 cycles. All reactions were performed in triplicate, and gene expression levels for *MARCKSL1* were calculated using the 2^−ΔΔCt^ formula.

### 2.6 In vitro assays for cell migration and invasion

Migration experiments were performed using 24‐well plate Transwell chambers (Corning). Cells (5 × 10^4^) were added to the upper chamber containing 100 μl of serum‐free RPMI‐1640 medium and 600 μl of RPMI‐1640 medium with 10% FBS was added to the lower chamber as a chemoattractant. After incubation for 48 h, cells that penetrated the membrane were fixed with methanol for 10 min and stained with 0.5% crystal violet, while cells remaining in the upper chamber were removed. The cells that invaded through the membrane were observed and counted within five random 100× fields under a microscope. With the exception of precoating with 30 μg of Matrigel (Corning), the invasion assays were performed using the same protocol as the migration assay. All experiments were performed in triplicate, and the results are presented as the mean ± SEM values.

### 2.7 Wound‐healing assay

Cells (5 × 10^5^ cells/well) were seeded in a 6‐well plate and cultured until confluence. A straight scratch was introduced with a sterile 200 μl tip to simulate a wound, which was washed twice with PBS and the medium was then changed to serum‐free RPMI 1640 medium. The images were taken at 0 h and 24 h. Experiments were performed in triplicate, and the results are presented as the mean ± SEM.

### 2.8 Cell proliferation assay

Cells (1.5 × 10^3^ cells/well) were seeded in 96‐well plates and cultured for 6 days. The viable cells were quantified every 24 h by adding 10 μl of CCK8 reagent (KeyGEN, Shanghai, China) to each well and incubating at 37°C for 1 h. Then, the absorbance was measured at 450 nm with a microplate reader (Molecular Devices). Each assay was carried out at least in triplicate.

### 2.9 Gelatin degradation assay

The gelatin degradation assay was performed as previously reported.^17^ In brief, the bottoms of glass dishes were first coated with 0.1% unconjugated gelatin (G1393, Sigma) and fluorescent gelatin (1:5 dilution of Oregon green 488 conjugated gelatin [G13186, Invitrogen]). Then the dishes were incubated with 5 mg/ml sodium borohydride for 15 min and 70% ethanol for 20 min. Before cell seeding, RPMI 1640 medium was added to dishes for 1 h at 37°C. The cells were incubated in the dishes at 37°C for an additional 48 h, fixed in 4% paraformaldehyde, permeabilized with 0.5% Triton X‐100, rinsed with PBS and blocked in 2% bovine serum albumin. Following primary antibody (anti‐Tks5/FISH, sc‐30, 122, Santa Cruz; anti‐cortactin) incubation, secondary antibodies labeled with Alexa Fluor 647 were added to the cells. Laser scanning confocal microscopy (PerkinElmer) was used to obtain fluorescence images.

### 2.10 Immunofluorescence staining

Immunofluorescence was performed as described previously.^17^ After fixation with 4% paraformaldehyde, permeabilization with 0.2% Triton X‐100 and blocking with 2% BSA, the cells were incubated with primary antibodies against MARCKSL1 (sc‐130,471, 1:50 diluted, Santa Cruz), F‐actin (Cat# 23127, 1:100 diluted, AAT Bioquest) and cortactin (Cat# 3503, 1:200 diluted, CST) at 4°C overnight. Following incubation with Alexa Fluor 594‐ or 488‐conjugated secondary antibodies (CST), cell nuclei were stained with DAPI. A laser scanning confocal microscope (PerkinElmer) was used to capture immunofluorescence images.

### 2.11 RNA‐sequencing analysis

Total RNA was extracted from KYSE140‐MARCKSL1‐KD (knockdown of MARCKSL1 by siRNA) and control cells by TRIzol reagent (Invitrogen) according to the manufacturer's instructions. RNA sequencing (RNA‐seq) was performed as described previously.^17^ The DNBSEQ platform (BGI, Beijing, China) was used for high‐throughput sequencing, and paired‐end sequencing reads of 150 nt were obtained.

### 2.12 TCGA data analysis

The RNA‐sequencing and clinical data of patients with ESCA in The Cancer Genome Atlas (TCGA) database (https://cancergenome.nih.gov/) were used to discover the alterations of MARCKSL1 in large cohort, in which the GEPIA2 platform (http://gepia2.cancer‐pku.cn/#index) was used to compare the mRNA expression levels of MARCKSL1 in ESCA tumors (*n* = 182) and normal tissues (*n* = 286).

### 2.13 Statistical analysis

Statistical analysis was performed using GraphPad Prism v8.0 software (GraphPad Software). All quantitative data are presented as the mean ± SEM. Differences between two groups were compared by two‐tailed Student's *t*‐tests or the Mann–Whitney rank sum test. Differences among three or more groups were compared by two‐tailed analysis of variance (ANOVA) followed by multiple comparisons tests. The qualitative data were compared using the chi‐square test. Survival analyses were performed using Kaplan–Meier curves combined with the log‐rank test. A *p* value less than 0.05 was considered statistically significant.

### 2.14 Study approval

This study was conducted in accordance with the guidelines of the Declaration of Helsinki, and it received approval. Exempt of informed consent was granted by the Institutional Review Board of the Ethics Committee of Cancer Hospital, Chinese Academy of Medical Sciences (ID: NCC1783).

## 3 RESULTS

### 3.1 Silencing of MARCKSL1 expression suppressed the invasion and migration of esophageal cancer cells in vitro

**FIGURE 1:**
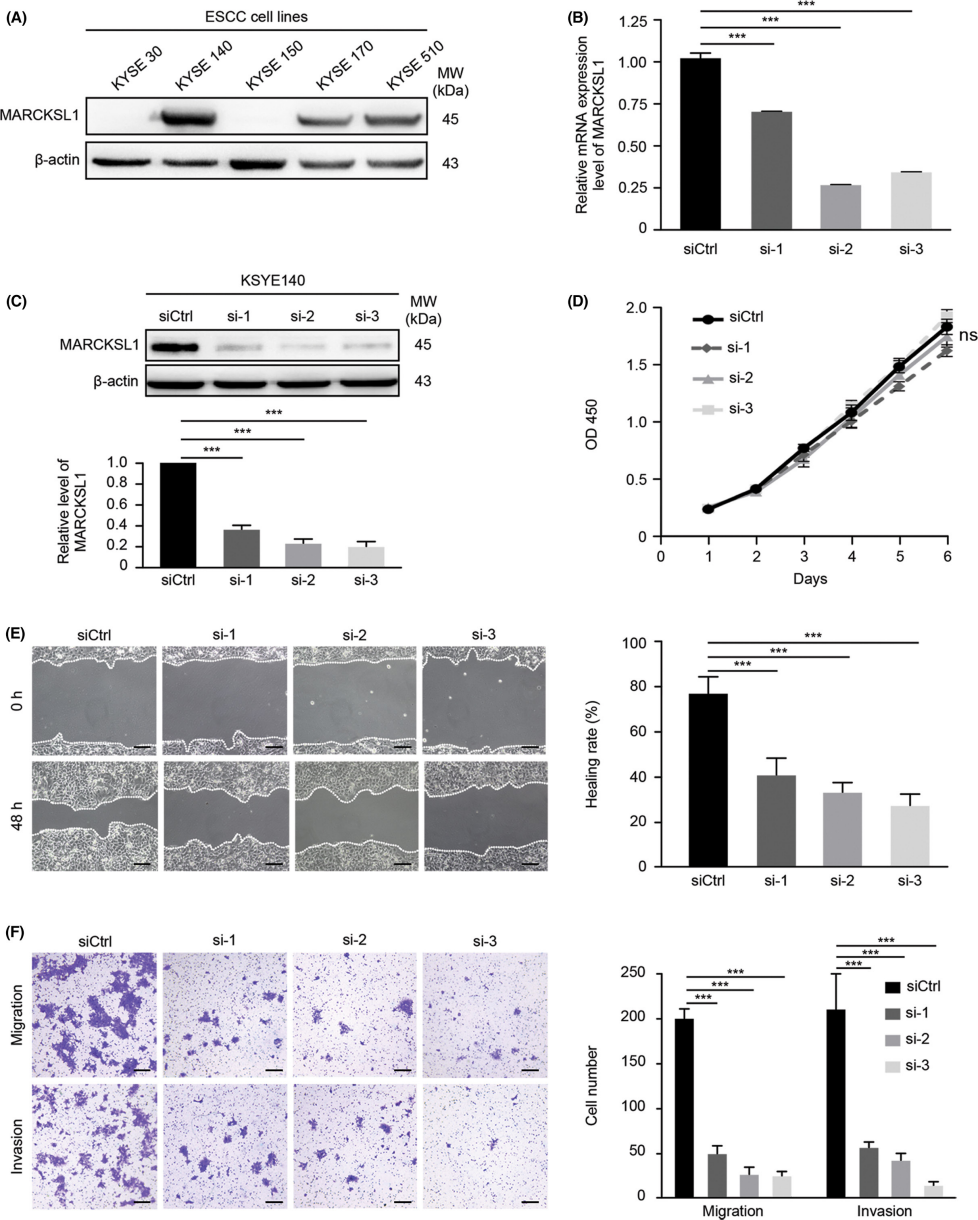
Silencing of MARCKSL1 expression suppressed the invasion and migration of esophageal cancer cells in vitro. (A) The protein levels of MARCKSL1 in ESCC cells were examined by western blot analysis. (B and C) ESCC KYSE140 cells were transiently transfected with siRNA targeting MARCKSL1 (siRNA‐1, ‐2 and ‐3) or scrambled siRNA control (siCtrl). The mRNA and protein levels of MARCKSL1 in siCtrl, siRNA‐1, siRNA‐2 and siRNA‐3 cells were tested by qPCR and western blotting, respectively. The mRNA and protein levels of MARCKSL1 were normalized to those of GAPDH and β‐Actin, with the fold changes relative to the control group. (D) The proliferation of siCtrl, siRNA‐1, siRNA‐2 and siRNA‐3 cells was measured by CCK8 assays. Two‐way ANOVA and a multiple comparisons test were used to determine the growth curve differences. (E, F) The effects of MARCKSL1 on the invasion and migration capacities of siCtrl, siRNA‐1, siRNA‐2 and siRNA‐3 cells were determined by the wound‐healing assays (E) and Transwell experiments (F), respectively. Scale bar: 100 μm. All experiments were performed in triplicate, and the representative data are presented as the mean ± SEM. Ns, not significant; ***p < 0.001.

To explore whether MARCKSL1 modulates ESCC progression, we first analyzed the effect of MARCKSL1 on ESCC progression in vitro. We tested the expression levels of MARCKSL1 in ESCC cells by western blotting and observed that MARCKSL1 expression was higher in KYSE140 cells, while KYSE30 and KYSE150 cells exhibited lower expression of MARCKSL1 (Figure 1A). Then, MARCKSL1 was knocked down in KYSE140 cells by siRNA, which was confirmed by qPCR and western blotting (Figure 1B,C). The CCK8 assay demonstrated that deletion of MARCKSL1 did not significantly suppress KYSE140 cell proliferation in a short period of time (Figure 1D). Compared with the negative control, the Transwell and wound‐healing assays both indicated that knockdown of MARCKSL1 markedly attenuated the cell invasion and migration capacity (Figure 1E,F). In total, these results revealed that downregulation of MARCKSL1 significantly suppressed the invasion and migration of ESCC cells.

### 3.2 Upregulation of MARCKSL1 expression enhanced the invasion and migration of ESCC cells in vitro

**FIGURE 2:**
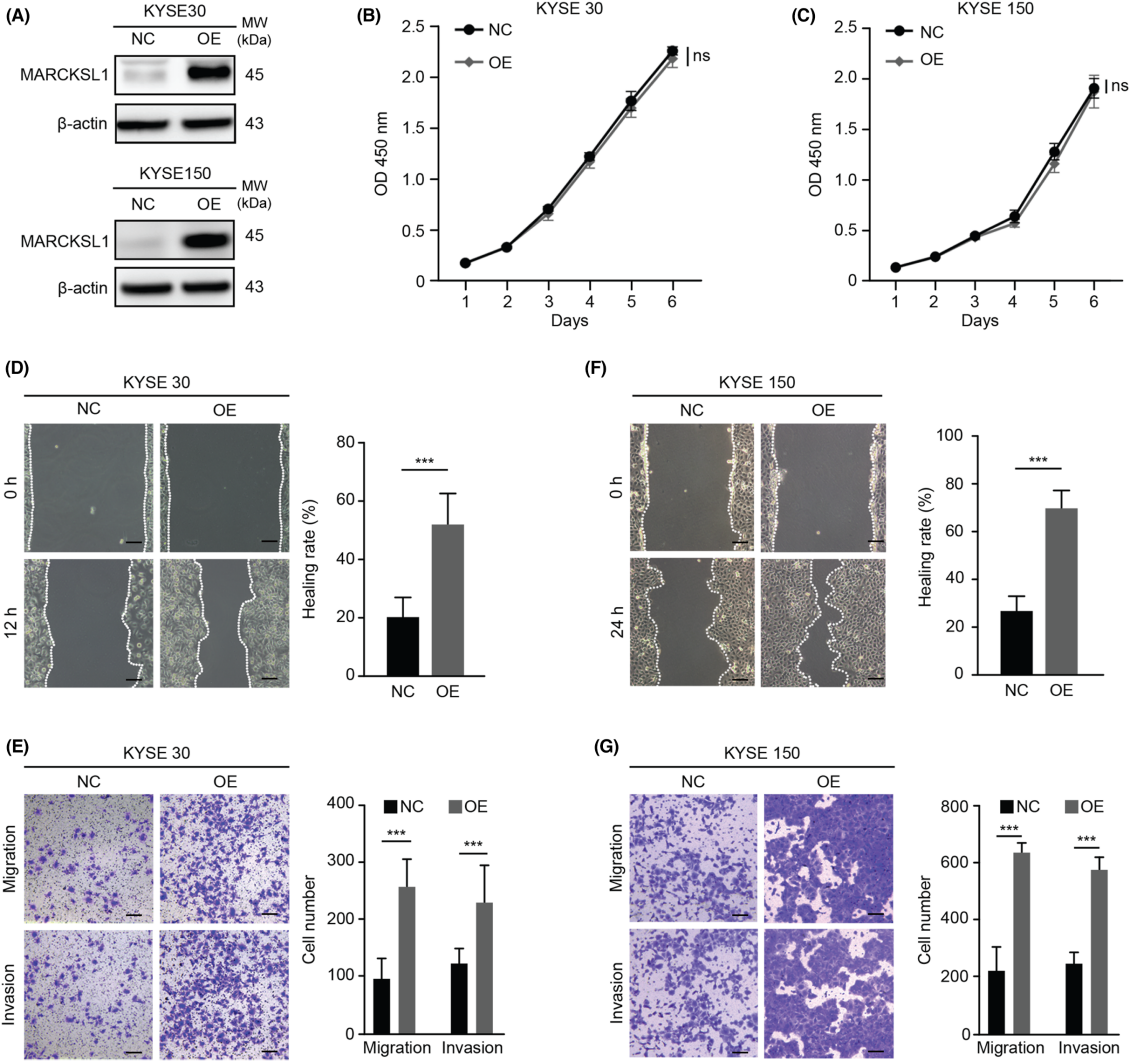
Upregulation of MARCKSL1 expression enhanced the invasion and migration of esophageal cancer cells in vitro. (A) Esophageal cancer KYSE30 and KYSE150 cells were transiently transfected with MARCKSL1 (OE) or an empty vector control (NC). The protein levels of MARCKSL1 in the OE and NC groups were assessed by western blot, and β‐Actin was used as a loading control. (B, C) The proliferation of KYSE30 (B) and KYSE150 (C) cells was measured by CCK8 assays. Using two‐way ANOVA followed by a multiple comparisons test, the differences among the growth curves were analyzed. (D‐G) The effects of MARCKSL1 on the invasion and migration capacities of KYSE30 and KYSE150 cells were detected via wound‐healing (D, F) and Transwell (E, G) assays. Scale bar: 100 μm. All experiments were performed in triplicate, and the representative data are presented as the mean ± SEM. Ns, not significant; ***p < 0.001.

KYSE30 and KYSE150 cells were then transiently transfected with MARCKSL1 (MARCKSL1 overexpression, OE) or an empty vector (as a negative control, NC), and MARCKSL1 overexpression was further verified in KYSE30 and KYSE150 cells by western blotting (Figure 2A). Consistent with the influence of MARCKSL1 knockdown on KYSE140 cell proliferation, MARCKSL1 overexpression did not enhance KYSE30 and KYSE150 cell proliferation in a short period of time (Figure 2B,C), while MARCKSL1 overexpression notably promoted migration and invasion (Figure 2D–G). Overall, it was demonstrated that MARCKSL1 was positively correlated with ESCC cell the metastasis in vitro.

### 3.3 MARCKSL1 regulated cell mobility and migration

**FIGURE 3:**
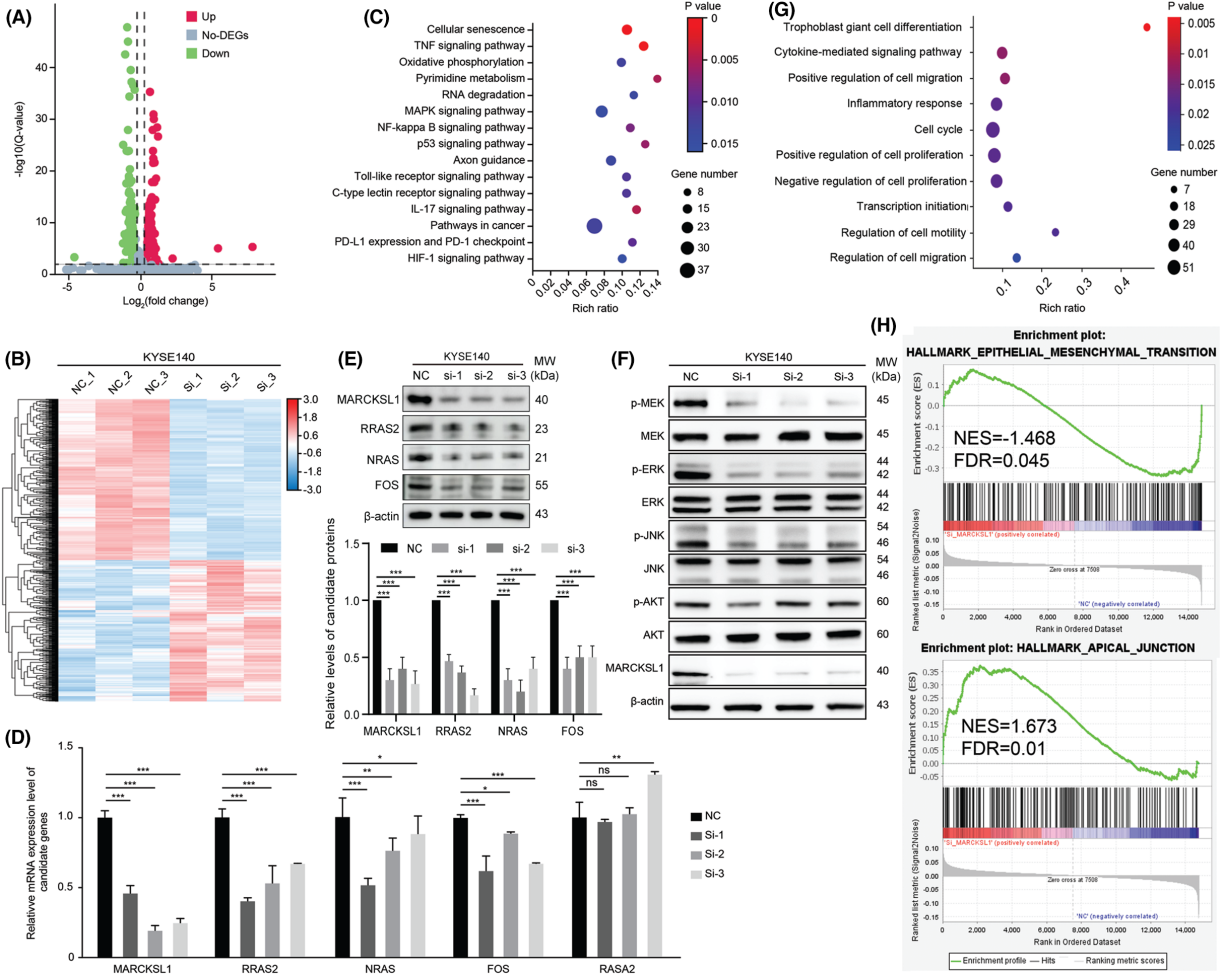
MARCKSL1 regulated cell mobility and migration as shown by RNA‐seq analysis. (A) A volcano plot of the distribution of all differentially expressed genes (DEGs) was constructed, mapping the 351 upregulated genes in red dots and the 449 downregulated genes in green dots after MARCKSL1 knockdown. (B) The heatmap of 800 DEGs is shown. Red denotes upregulation, and blue represents downregulation. (C) KEGG pathway analysis among the 800 DEGs was performed via DAVID. The top 15 enriched pathways are shown. (D and E) The mRNA (D) and protein (E) expression levels of representative genes associated with the MAPK signaling pathway were confirmed by qPCR and western blot analysis in KYSE140 cells. The representative data are presented as the mean ± SEM from independent triplicate experiments. Ns, not significant; *p < 0.05; **p < 0.01; ***p < 0.001. (F) Western blotting showed marked decreases in the phosphorylation of MEK, ERK and JNK in KYSE140 cells after MARCKSL1 knockdown . (G) Gene Ontology (GO) analysis based on 800 DEGs was performed by DAVID. The top 10 enriched pathways are shown, including cell differentiation, cell migration, proliferation and motility. (H) Gene Set Enrichment Analysis (GSEA) enrichment profiles from RNA‐sequencing reveal that MARCKSL1 is significantly associated with epithelial‐mesenchymal transition and apical junction.

To better understand the underlying mechanisms by which MARCKSL1 mediates ESCC progression, transcriptome profiling was conducted by RNA sequencing (RNA‐seq) analysis after knockdown of MARCKSL1 by siRNA in KYSE140 cells. A total of 800 differentially expressed genes (DEGs) were identified compared to the negative control with the following screening criteria: *p* ≤ 0.01 and fold change ≥1.2 or ≤0.83, including 351 upregulated and 449 downregulated genes (Figure 3A,B; Table S1). Next, KEGG pathway analysis was performed among 800 DEGs and showed that the DEGs were markedly enriched in cellular senescence and the TNF, MAPK and NF‐kappa B signaling pathways (Figure 3C). Then, we investigated the effect of MARCKSL1 on the MAPK signaling pathway and found that deletion of MARCKSL1 significantly reduced both the mRNA (Figure 3D) and protein (Figure 3E) expression levels of RRAS2, NRAS and FOS, which are pivotal hubs of the MAPK signaling pathway. Furthermore, western blot analysis indicated that knockdown of MARCKSL1 inhibited the phosphorylation of MEK, JNK and ERK (Figure 3F), while the expression levels were not affected, suggesting that knockdown of MARCKSL1 could significantly inactivate the MAPK signaling pathway. In addition, GO analysis was performed and suggested that the 800 DEGs were markedly enriched in cell differentiation, cytokine‐mediated signaling pathway, cell migration and motility (Figure 3G), consistent with the phenotypic experimental assay that showed upregulation of MARCKSL1 expression enhanced ESCC cell invasion and migration in vitro. Importantly, GSEA revealed that knockdown of MARCKSL1 by siRNA significantly enhanced the epithelial phenotype (Figure 3H) for instance, depletion of MARCKSL1 markedly promoted the apical junction of ESCC cells. Taken together, these results support that MARCKSL1 mediates the mobility and migration of esophageal cancer cells.

### 3.4 MARCKSL1 promotes invasion and metastasis in ESCC through increased invadopodia formation and ECM degradation

**FIGURE 4:**
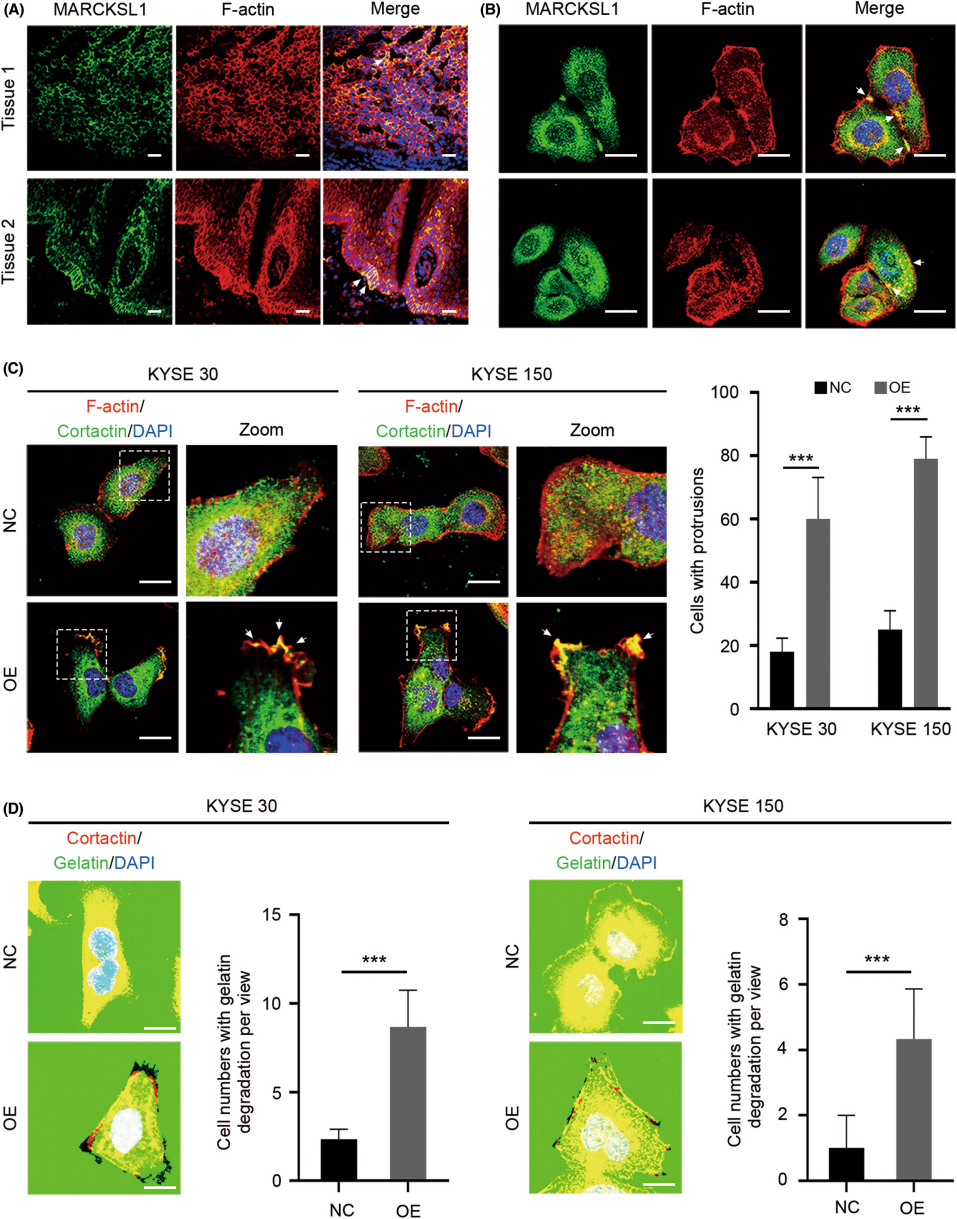
MARCKSL1 Promotes invasion and metastasis in ESCC through increased invadopodia formation and ECM degradation. (A) The colocalization of MARCKSL1 (green) and F‐Actin (red) was observed by immunofluorescence staining in ESCC tissues. The colocalization of MARCKSL1 and F‐Actin is indicated by white arrows. DAPI (blue) staining was used to visualize nuclei. Scale bar: 100 μm. (B) The colocalization of MARCKSL1 (green) and F‐Actin (red) was visualized by immunofluorescence staining in KYSE30 cells. The colocalization of MARCKSL1 and F‐Actin is indicated by white arrows. DAPI (Blue) was used for nuclear staining. Scale bar: 30 μm. (C) Left panel: MARCKSL1 Overexpression (OE) facilitated the aggregation of colocalization between F‐Actin and cortactin to form invasive protrusions toward the edge of the cell membrane in KYSE30 (left) and KYSE150 (right) cells compared with the negative control (NC). F‐Actin (red) and cortactin (green) were visualized under a confocal microscope after immunofluorescence staining. DAPI (blue) was used to stain nuclei. Scale bar: 30 μm. Right panel: The quantitative analysis of invasive protrusions on the edge of the cell membrane between OE and NC cells. (D) A gelatin degradation assay indicates that overexpression of MARCKSL1 (OE) promotes extracellular matrix (ECM) degradation on invasive protrusion toward the edge of the cell membrane in KYSE30 (left) and KYSE150 (right) cells. The numbers of cells degrading gelatin per view were calculated. Scale bar: 30 μm; ***p < 0.001.

The metastatic potential of tumor cells partially depends on invasion through the extracellular matrix. MARCKSL1 has been shown to interact with F‐actin to determine actin stability and cell migration.^5^, ^6^ To further determine whether MACKSL1 promotes ESCC progression by interacting with F‐actin, an immunofluorescence assay was carried out. Endogenous MARCKSL1 colocalized with F‐actin in ESCC tissues (Figure 4A) and cells (Figure 4B) both in vitro and in vivo. It has been clearly demonstrated that F‐actin is implicated in the formation of invadopodia,^18^ so we initially speculated that MARCKSL1 might be involved in invadopodia formation to regulate ESCC progression. Cortactin is an important marker of invadopodia.^18^ Cortactin expression in the membranes of cells overexpressing MARCKSL1 (MARCKSL1‐OE) was analyzed by immunofluorescence staining. The results suggested that MARCKSL1 overexpression notably promoted the colocalization of F‐actin and cortactin in the membrane (Figure 4C), especially at the leading edge of migrating cells, demonstrating that MARCKSL1 mediated the formation of invadopodia. Moreover, consistent with these increases in the number of invadopodia‐forming cells, overexpression of MARCKSL1 also increased the area of gelatin degraded per cell (Figure 4D). These data further confirmed that overexpression of MARCKSL1 significantly promotes invadopodia formation and ECM degradation.

### 3.5 High MARCKSL1 expression correlated with metastasis and poor prognosis in patients with ESCC

**FIGURE 5:**
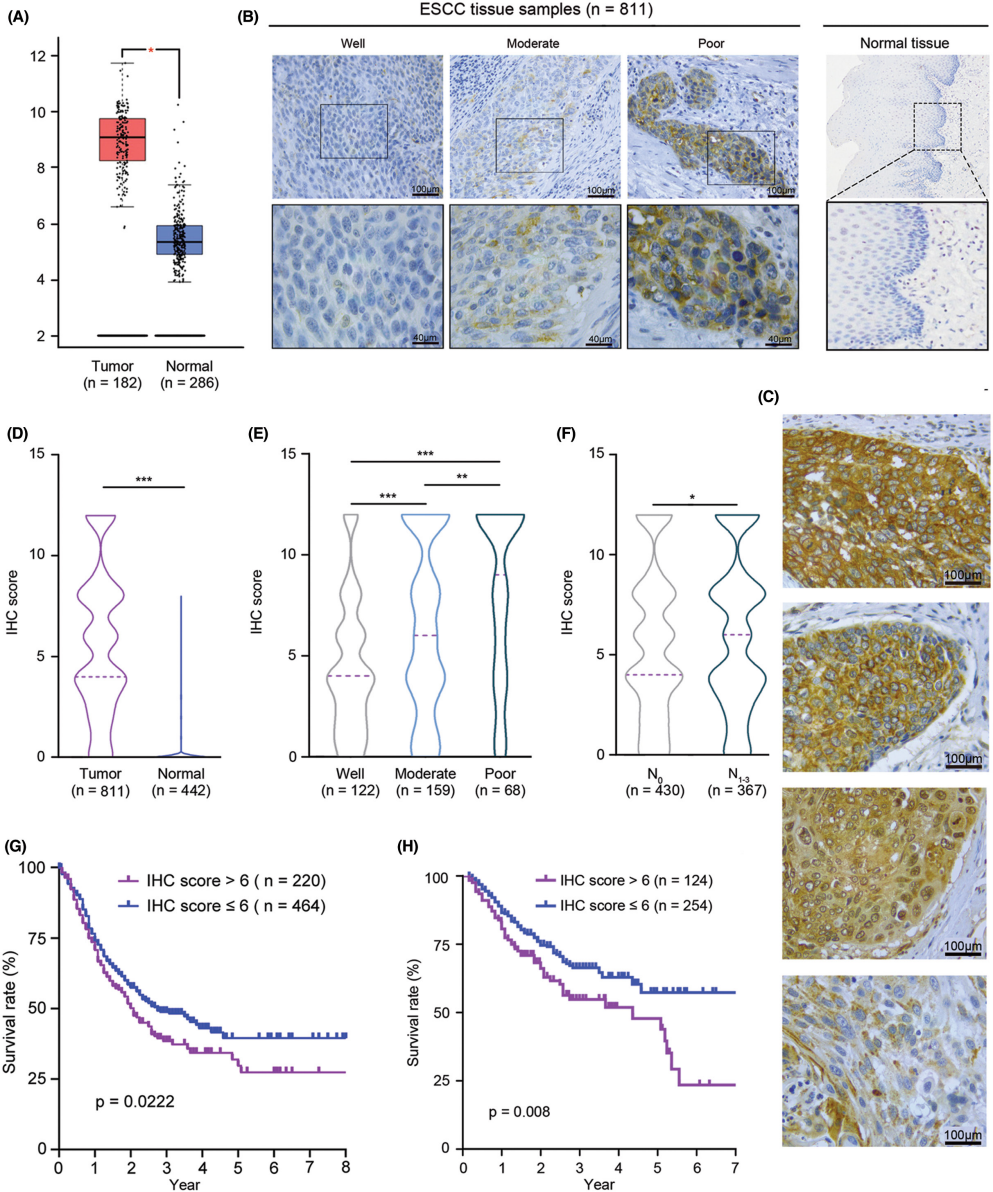
MARCKSL1 overexpression correlated with tumor metastasis and worse prognosis in ESCC patients. (A) The mRNA levels of MARCKSL1 in esophageal cancer (*n* = 182) and normal esophageal tissues (*n* = 286) were analyzed from TCGA databases. (B) The expression levels of MARCKSL1 in ESCC specimens with well, moderate and poor differentiation grades (left) and adjacent nontumorous (right) tissues were measured by immunohistochemical staining. Representative regions (black frame) at low magnification (400×, top) are amplified at high magnification (1000×, bottom). Scale bars: 40 or 100 μm. (C) Representative images of MARCKSL1 with strong membrane, granular and diffuse staining and weak diffuse staining in ESCC tissues. Scale bars: 100 μm. (D) The violin plot of the IHC scores for MARCKSL1 in ESCC tissues (*n* = 811) and adjacent nontumorous epithelia (*n* = 442) is shown. (E) The IHC scores of MARCKSL1 in ESCC tissues with well (*n* = 122), moderate (*n* = 159) and poor (*n* = 68) differentiation grades are shown. (F) The IHC score of MARCKSL1 in tumor tissues with (*n* = 430) or without lymph node metastasis (*n* = 367) are shown. (G) Kaplan–Meier survival curves were constructed for ESCC patients with high (IHC score >6, *n* = 220) and low (IHC score ≤6, *n* = 464) MARCKSL1 expression. (H) Kaplan–Meier survival curves were constructed for ESCC patients with negative lymph node metastasis and high (IHC score >6, *n* = 124) and low (IHC score ≤6, *n* = 254) MERCKSL1 expression. *p < 0.05; **p < 0.01; ***p < 0.001.

To explore whether MARCKSL1 modulates tumor progression in patients with esophageal cancer, we analyzed gene expression from The Cancer Genome Atlas (TCGA) database and obtained RNA‐seq fragments per kilobase million (FPKM) values from the TCGA Genomic Data Commons (GDC) portal. Then the log2 transformed values were calculated. The mRNA level of MARCKSL1 in esophageal carcinomas (*n* = 182) was notably higher than that in adjacent noncancerous tissues (*n* = 286) (Figure 5A). Then, the expression of MARCKSL1 was assessed in tissue microarrays using an immunohistochemical assay. The expression of MARCKSL1 was significantly increased in ESCC tumor tissues (*n* = 811) compared to adjacent nontumorous tissues (*n* = 442) (Figure 5B–D). Moreover, the expression of MARCKSL1 from fresh tissue resection in patients with ESCC (*n* = 40) was also confirmed to be markedly increased. To further investigate whether MARCKSL1 regulates ESCC progression, correlation analysis between MARCKSL1 expression and clinicopathological characteristics was performed. The expression of MARCKSL1 was significantly correlated with differentiation grade, and ESCC patients with poor differentiation had higher expression of MARCKSL1 (Figure 5E). Intriguingly, the expression of MARCKSL1 was positively correlated with lymph node metastasis (Figure 5F), implying that MARCKSL1 promotes ESCC invasion. Furthermore, it was demonstrated that ESCC patients with high expression levels (*n* = 220, IHC score >6) of MARCKSL1 had a worse survival rate than those with low expression levels (*n* = 464, IHC score ≤6) (*p* = 0.0222) by Kaplan–Meier survival analysis (Figure 5G). MARCKSL1 has a significant prognostic value in lymph node‐negative breast cancer.^19^ To explore whether the protein level of MARCKSL1 is related to prognosis in lymph node‐negative ESCC patients, Kaplan–Meier survival analysis was also performed in ESCC patients with lymph node‐negative ESCC (*n* = 378) and showed that higher MARCKSL1 expression was markedly correlated with a poor survival rate (Figure 5H). However, no correlations were found between MARCKSL1 expression and other clinicopathological characteristics including age, sex, tumor length, tumor location and TNM stage (Table 1). Briefly, our results suggested that MARCKSL1 was involved in ESCC progression, especially ESCC with lymph node metastasis and poor prognosis.

**TABLE 1:** MARCKSL1 expression in relation to the clinicopathological characteristics of 811 ESCC patients

Characteristics	Cases (n)	MARCKSL1 expression		
		Low (n)	High (n)	*p*
Age (year)				0.084
≤60	294	193	101	
>60	517	321	196	
Sex				0.555
Male	608	385	223	
Female	203	129	74	
Differentiation				<0.0001
Well	122	98	24	
Moderate	159	89	70	
Poor	68	24	44	
Unknown	462	303	159	
Tumor location				0.954
Upper	23	12	11	
Middle	159	90	69	
Lower	65	34	31	
Unknown	564	378	186	
T stage				0.398
T1‐2	187	117	70	
T3‐4	563	359	204	
Unknown	61	38	23	
Lymph node metastasis				0.012
N0	430	289	141	
N1‐3	367	217	150	
Unknown	14	8	6	
TNM stage				0.048
I‐II	402	272	130	
III‐IV	351	206	145	
Unknown	58	36	22	

*Note*: The *p* value was calculated by the chi square test.

## 4 DISCUSSION

Esophageal squamous cell carcinoma (ESCC) is one of the most common malignancies in the world,^1^ but it remains poorly understood how ESCC cells metastasize from the primary tumor to surrounding or even distant lymph nodes. Here, we identified and demonstrated that MARCKSL1 accelerates ESCC cell migration and invasion by wound‐healing and Transwell assays, indicating that MARCKSL1 participates in ESCC progression, while MARCKSL1 has no impact on cell proliferation in a short period of time.

To further reveal the mechanistic basis by which MARCKSL1 enhances cell migration and invasion, RNA‐seq analysis was performed, and we observed DEGs after knockdown of MARCKSL1 that were markedly enriched in the TNF, MAPK and NF‐kappa B signaling pathways, results that were partially validated by qPCR and western blotting (Figure 3C–E). Importantly, in line with previous reports,^20^ which showed that MARCKSL1 modulates the epithelial‐mesenchymal transition (EMT) by affecting the expression of EMT‐associated proteins, RNA‐seq analysis also indicated that knockdown of MARCKSL1 significantly facilitated the epithelial phenotype (Figure 3H); for instance, depletion of MARCKSL1 markedly promoted the apical junction of ESCC cells. Interestingly, although MARCKSL1 is directly phosphorylated by JNK on C‐terminal residues,^5^ which induces F‐actin bundle formation and stabilization, thereby reducing actin plasticity and restricting cell movement, western blot analysis indicated that knockdown of MARCKSL1 could inhibit JNK phosphorylation (Figure 3F), implying that there is negative feedback regulation between MARCKSL1 and JNK phosphorylation cascades to mediate cell mobility. In addition, RNA‐seq showed both positive and negative regulation of cell proliferation after knockdown of MARCKSL1 (Figure 3G), which likely explains why MARCKSL1 does not influence cell proliferation.

Previous reports have shown that MARCKSL1 potentiates actin polymerization and cytoskeletal organization in the early development of the nervous system.^5^, ^6^, ^8^, ^9^ Moreover, phosphorylation of MARCKSL1 also stabilizes and increases the bundling of F‐actin, resulting in alterations in filopodium morphology and dynamics, hence modulating cell migration.^5^ Since F‐actin‐rich membrane structures at the leading edge of migrating cells could form invadopodia that engage in proteolytic activity and degrade the surrounding extracellular matrix (ECM) to facilitate tumor cell penetration through the epithelial and endothelial basement membranse and because there is colocalization between MARCKSL1 and F‐actin at the leading edge of the cell, we speculate that MARCKSL1 participates in the formation of invadopodia to modulate cell mobility. To this end, an immunofluorescence assay was performed, and we found that endogenous MARCKSL1 colocalized with F‐actin in ESCC cells. Moreover, overexpression of MARCKSL1 notably promoted the colocalization between cortactin and F‐actin on the leading edge of migrating cells and increased ECM degradation, supporting that MARCKSL1 mediated ESCC migration and invasion through invadopodia formation.

A previous systematic and integrative ‘omics’ strategy identified that MARCKSL1 was upregulated in ESCC tissues and that MARCKSL1 augments ESCC cell mobility in vitro,^15^ but it remains unclear whether MARCKSL1 expression is notably associated with the clinicopathological characteristics of patients with ESCC. Therefore, an immunohistochemical assay was performed, and as described in previous reports,^19^ we observed that MARCKSL1 staining showed strong membrane, granular and diffuse staining, suggesting that MARCKSL1 has different regional expression patterns and potentially distinctive functions in different regional localizations. Moreover, MARCKSL1 protein levels were significantly increased in ESCC tumor tissues (*n* = 811) compared to adjacent esophageal epithelia (*n* = 442). Excitingly, the MARCKSL1 protein level was markedly connected with differentiation grade, and ESCC patients with poor differentiation had higher expression of MARCKSL1 (Figure 5E). In addition, the protein level of MARCKSL1 was positively correlated with lymph node metastasis (Figure 5F), supporting that MARCKSL1 promotes ESCC cell mobility, and ESCC patients with high expression levels of MARCKSL1 had a worse survival rate than those with low expression levels (Figure 5G). It has been shown that MARCKSL1 has a significant prognostic value in lymph node‐negative breast cancer,^14^, ^18^ hence, Kaplan–Meier survival analysis was performed in ESCC patients with lymph node‐negative disease, which showed that higher MARCKSL1 expression was markedly correlated with a poor survival rate (Figure 5H). Taken together, our results support that MARCKSL1 is involved in ESCC progression in vivo, especially in ESCC patients with lymph node metastasis and poor prognosis.

In conclusion, we demonstrated that upregulation of MARCKSL1 markedly promoted cell migration and invasion and that MARCKSL1 affected the formation of invadopodia and ECM degradation to promote ESCC metastasis. In addition, a higher protein level of MARCKSL1 was positively correlated with lymph node metastasis, and ESCC patients with high expression levels of MARCKSL1 had a worse survival rate, thereby providing an applicable prognosticator and a potential therapeutic target for ESCC patients.

## AUTHORS' CONTRIBUTIONS

YZ, XX and LT performed the main experiments and obtained and analyzed the data. FL, XX, YS and HL performed IHC staining and data analysis. YZ and LT wrote the manuscript draft. XZ and LT analyzed the data and critically revised the manuscript. XZ and YM conceived, designed and coordinated the experiments. All authors reviewed and approved the manuscript for submission.

## FUNDING INFORMATION

National Natural Science Foundation of China, Grant/Award Number: 81872033 and 82,073,327; National Key R & D Program of China, Grant/Award Number: 2018YFC1313101 and 2016YFC0901403; CAMS Innovation Fund for Medical Sciences, Grant/Award Number: 2016‐I2M‐1‐001, 2019‐I2M‐1‐003 and 2021‐I2M‐1‐066.

## CONFLICT OF INTEREST

The authors declare no conflict of interest.

## ETHICS STATEMENT

This study was approved by the Institutional Review Board of the Ethics Committee of Cancer Hospital, Chinese Academy of Medical Sciences (ID: NCC1783) and performed in accordance with the guidelines of the Declaration of Helsinki.

## Supporting information


Table S1

## Data Availability

The data presented in this study are available from the corresponding authors on request.
